# Integrating data from multiple sources for insights into demographic processes: Simulation studies and proof of concept for hierarchical change-in-ratio models

**DOI:** 10.1371/journal.pone.0194566

**Published:** 2018-03-29

**Authors:** Erlend B. Nilsen, Olav Strand

**Affiliations:** Norwegian Institute for Nature Research, Torgarden, Trondheim, Norway; Oregon State University, UNITED STATES

## Abstract

We developed a model for estimating demographic rates and population abundance based on multiple data sets revealing information about population age- and sex structure. Such models have previously been described in the literature as change-in-ratio models, but we extend the applicability of the models by i) using time series data allowing the full temporal dynamics to be modelled, by ii) casting the model in an explicit hierarchical modelling framework, and by iii) estimating parameters based on Bayesian inference. Based on sensitivity analyses we conclude that the approach developed here is able to obtain estimates of demographic rate with high precision whenever unbiased data of population structure are available. Our simulations revealed that this was true also when data on population abundance are not available or not included in the modelling framework. Nevertheless, when data on population structure are biased due to different observability of different age- and sex categories this will affect estimates of all demographic rates. Estimates of population size is particularly sensitive to such biases, whereas demographic rates can be relatively precisely estimated even with biased observation data as long as the bias is not severe. We then use the models to estimate demographic rates and population abundance for two Norwegian reindeer (*Rangifer tarandus*) populations where age-sex data were available for all harvested animals, and where population structure surveys were carried out in early summer (after calving) and late fall (after hunting season), and population size is counted in winter. We found that demographic rates were similar regardless whether we include population count data in the modelling, but that the estimated population size is affected by this decision. This suggest that monitoring programs that focus on population age- and sex structure will benefit from collecting additional data that allow estimation of observability for different age- and sex classes. In addition, our sensitivity analysis suggests that focusing monitoring towards changes in demographic rates might be more feasible than monitoring abundance in many situations where data on population age- and sex structure can be collected.

## Introduction

Due to their popular status as game animals, efficient management of ungulate populations often requires detailed knowledge about their demography and abundance [1]. When environmental conditions varies, this will often result in demographic rate variation [2] causing the finite population growth rate (λ) to fluctuate in time and space. For spatially closed populations, demographic rate variation, together with fluctuations in the age structure [3, 4], are the ultimate source of variation in λ. As a result, the tradition of studying the link between demography and population state has a long history in large mammal research [5–8]. To this end, a wide range of methods and study approaches have been taken in order to estimate demographic rates and population sizes. For instance, for many northern and temperate ungulates, radio telemetry has been used to estimate survival probabilities [9, 10] and recruitment rates [11, 12]. In addition, great methodological advances in the field of capture-mark-recapture (CMR) studies have put much focus on the benefits and potential for insight from marked individuals [13] even if they are not radio collared. While radio collaring has been used mainly for research purposes, the opportunities offered by CMR methods has been gradually implemented in monitoring programs [14, 15]. In particular, many large carnivore monitoring programs are currently implementing CMR analysis based on non-invasive sampling (e.g. of scats; see [16]). Such methods have, however, been far less utilized to monitor ungulate populations, partly because ungulates usually live at far higher densities and because available funds for monitoring are limited. These methods, which are both time consuming and often conflict with the limited funds that are available for wildlife management, generally make them less applicable in real life situations. There is thus a dire need to develop new cost-efficient tools that both meet the rigor expected from any mature monitoring scheme (e.g. accounting for detection probabilities that might vary in time and space; [17]) and at the same time are applicable and sustainable over long time periods across large landscapes.

Wild mountain reindeer (*Rangifer tarandus*) are endemic to Norway, and are of national and international conservation interest. Reindeer formerly occupied large continuous areas of mountain landscape, but roads, railroads, mountain cabins and other infrastructure have fragmented the landscape and impeded movements across the landscape [18, 19]. Consequently, wild reindeer in Norway are currently distributed as 23 isolated populations with many of the former migration routes being abandoned [18]. As a result of these landscape alterations many sub-populations also inhabit areas that are less suitable for either summer or winter residency [19]. Further, in addition to being an iconic species in Norwegian mountains, reindeer are harvested annually for sport and population management in most or all sub-populations [20]. These factors together make reindeer management a particularly sensitive natural resource issue. This situation afforded us an interesting case study of a managed ungulate species for which detailed knowledge is needed for effective population management.

During the last decade, hierarchical statistical models [21] that integrate information from multiple data sources [22–24] have been used to estimate abundance and vital rates from animal populations. In the classical integrated population model, CMR-data is typically modelled simultaneously with count data, to estimate demographic processes (i.e. survival probabilities and recruitment rates) as well as population size and rate of change [21, 25]. In other cases, methods frequently cited in the wildlife management literature (e.g. virtual population analysis; [26]) have been extended and accommodated to allow for joint modelling of complementary data sources. For instance, the use of auxiliary data has been used to increase precision and reliability of traditional age-at-harvest models for black bear (*Ursus americana*) in Minnesota, USA [22], and for greater sage-grouse (*Centrocercus urophasianus*) in Oregon, USA [27]. Despite increased use of integrated population models, some approaches, including change-in-ratio models (see also [26] and references therein, and [28]), have received relatively little attention. The main idea behind these estimators is that the population sex- and age structure (or some component of that) is sampled before and after some main source of mortality occurs [28]. For hunted wildlife population, a natural and often used mortality pulse is the annual harvest which is often confined to a limited time period. When the number, sex and age-class of harvested animals is also known, both survival rates and the abundance can be estimated under a given set of assumptions [26, 29].

Here we cast the change-in-ratio models in an explicit hierarchical modelling framework within a Markov Chain matrix population model and estimate parameters of interest based on Bayesian inference. First, we made extensive simulations to assess the robustness of the estimators to deviations from model assumptions. Next, we used the models to estimate parameters of interest from two wild reindeer populations, taking advantage of the typical multi-year monitoring programs for wildlife populations in Norway [20].

## Materials and methods

### Setting and survey protocols

The general sampling scheme and timing of events underlying the models presented here resembles those of a traditional three-sample change in ratio sampling scheme [26, 28]. One crucial extension is that we explicitly formulated the estimators through a Markov Chain model, where the parameter estimates not only results from the current year’s survey but also results from the Markov Chain properties of the model. The modelling is based on the following distinct data collection events:

Prior to harvest season (PRE), age and sex composition of the population is assessed. In our specific case, however, only a certain segment of the population is sampled as the main purpose of the pre-harvest surveys is to determine the annual calf production. Further, due to morphological similarities, only two classes of individuals are recognized: i) calves, and ii) a composite class consisting of adult females and yearlings of both sexes. Depending on the timing of this sampling event, the resulting estimators might represent a combination of fecundity *f* and juvenile summer survival φ_1_. In our case, when the pre-harvest survey occurs a short time after calving (between June 20^th^ and July 20^th^), the two rates will appear as distinct parameters in our estimators, and will both be estimable. The calf to female ratio is estimated based on visual examination of aerial photographs taken during summer surveys. Summer surveys are done in late June or in the first two weeks of July. A small fixed wing aircraft is used flying transects with overlapping visibility. All encountered herds are photographed and detected animals are later assigned to categories: calf, yearling and female and male 2 years and older (see [20] for further details).

Harvest (HARV) takes place as a pulse event (starting August 20^th^ and ending September 20^th^), removing a known number of individuals with known sex and age. Exact age is not required if the age classes match other available population data. described later. If the exact harvest off-take is not known a probabilistic model for the harvest process could possibly be constructed given that covariates known to be correlated to actual harvest is available (e.g. quota size and hunting effort).

After the harvest season (POST), the age- and sex structure of the population is again sampled. Sampling takes place in the first week after the harvest season (September 20^th^ to the first week of October). In our specific case, three classes of individuals are recognized: i) calves (of both sexes), ii) females (yearlings and older) and iii) males (yearlings and older). The surveys take place annually during the rut in October. This is a time when sexual segregation breaks down and all animals are aggregated in mixed sex groups. Surveys are conducted as ground surveys using a spotting scope. Herds of differing sizes are observed at distances of 50–300 m, and each animal is classified to age and sex by their body size, antler development, and visible genitals. Pre-harvest surveys, harvest data and post-harvest surveys are included in a national ungulate monitoring program financed by the Norwegian environment agency [30]. Data from the program can be downloaded at: http://www.hjorteviltregisteret.no/Villrein and at https://www.ssb.no/jord-skog-jakt-og-fiskeri/statistikker/reinjakt.

In addition, the total population size (TOT) is assessed during mid-winter (January through March) in both populations (see [20] for a similar sampling regime in another Norwegian wild reindeer population). The objective is to obtain a minimum count of the reindeer in the area each year. Surveys are conducted by flying transects with fixed-wing aircraft, and all reindeer groups that are seen are approached and photographed. Group size is then determined from the photographs. However, as it is well known that population estimates which are not based on a sampling scheme that allow observation probability to be estimated separately are often biased low [14], we considered models that both include and exclude this data set.

While the sampling events described above matches that of a more traditional three-sample change-in-ratio model [26], there are at least four factors that make our approach novel. First, we used time series data allowing the full temporal dynamics to be modelled. Second, we cast the model in an explicit hierarchical modelling framework, allowing the separation of the observation process from the state process [21]. Third, we estimate parameters based on Bayesian inference allowing us to exploit the great flexibility of the BUGS language. Fourth, the fact that the age- and sex structure of the population is differently represented in the different data sets complicates the modeling slightly. For instance, while yearling males are included in the female/yearling state in the PRE data sat, they are included in the male state in the POST data set and represent a single state in the HARV data set. While such a sampling scheme would make the traditional estimators less useful, our explicit formulation of the model as a Markov chain matrix population model allow us to estimate the hidden states and thus utilize the data described above.

### Population model specification

To utilize the four data sets described above in a joint analysis, we combined the datasets in a common population dynamic model. All data sets except the total surveys (TOT) includes information about the age and sex structure of the population, and thus serves as basis for constructing age- and sex structured population models [31–33]. Such models have proven useful when modeling the dynamics of long-lived species, as their demographic rates are typically strongly age-dependent [34, 35]. Different age classes can differ in their contribution to inter-annual changes in population increment and fluctuations in population structure might complicate the dynamical patterns further [3].

Population modelling require a proper specification of the presumed time schedule. There are two broad time schedules used in matrix population models for organisms with discrete breeding seasons, namely a pre-breeding or post-breeding time schedule [33]. In a post-breeding time schedule, the population state is updated immediately after birthing season and the youngest age group included in the population vector is the newly born individuals. Employing a pre-breeding model, the population vector is updated immediately before the birthing season, and the youngest age class is the almost one year old recruits. However, alternatives are possible, and sometimes a more detailed description of the annual cycle is needed with sub-processes describing the transition from one state vector to the next [32, 36]. In our case, the different data sets are collected at different times through the year, and we exploit the benefits of specifying the transition from year *t-1* to *t* through a series of two sub-process. First, we assume that the annual cycle starts just prior to the harvest season, and represents the population vector at that time by *N*_*t*_. Note that our approach approximates a mid-point between a post-breeding and a pre-breeding model, with the youngest age in the population vector including ca. 3 month old calves. We then assume that harvest occurs as a pulse, and reduces the population vector to *X*_*t*_ by removing a known number of individuals with known sex- and age. The harvest vector is represented by *H*_*t*_. Such a partitioning of the annual cycle allows us both to model surveys taken at different times of the year and to properly account for stochastic and deterministic properties of the state process.

We defined *N*_*t*_ (the population vector prior to harvesting in fall in year *t*) to be represented by a vector containing three age classes indexed as calves (c), yearlings (y) and adults (ad)) and two sexes indexed as males (M) and females (F):

$$N_{t}=\left[\begin{array}{c} \begin{array}{c} N_{c}^{F}\\ N_{y}^{F} \end{array}\\ N_{ad}^{F}\\ \begin{array}{c} N_{c}^{M}\\ N_{y}^{M}\\ N_{ad}^{M} \end{array} \end{array}\right]$$ (1)

We then assume that harvest occur as a pulse at the beginning of the time step, and represents the post-harvest population size in year *t* with *X*_*t*_:

$$X_{t}=N_{t}-H_{t}$$ (2)

The age- and sex distribution of animals harvested in year *t* is then represented by the vector *H*_*t*_. Note that our approach assumes that harvest bag statistics are reported without error, which is justified in our case because it is mandatory for hunters to report age class and sex of harvested reindeer to the management authorities [20]. We use only three age classes in our model (calves, yearlings and older, respectively) and the error should be minimal.

To transfer the population in year *t* to year *t+1*, we multiplied the post-harvest population vector in year *t* (*X*_*t*_) with the transition matrix **A**_t_:

$$N_{t+1}=X_{t}+A_{t}$$ (3)

The projection matrix **A**_t_ is a matrix with *i* columns and *j* rows. Each matrix element α_ij_ in **A**_t_ specifying the contribution from animals in class *i* in year *t* to class *j* in year *t+1* [33, 37]. Our transition matrix, transferring the population vector from *X*_*t*_ to *N*_*t+1*_ takes the form of a 6x6 square matrix:

$$A_{t}=\left[\begin{array}{cccccc} 0 & \phi_{2}\phi_{1}\frac{f}{2} & \phi_{2}\phi_{1}\frac{f}{2} & 0 & 0 & 0\\ \phi_{2} & 0 & 0 & 0 & 0 & 0\\ 0 & \phi_{2} & \phi_{2} & 0 & 0 & 0\\ 0 & \phi_{2}\phi_{1}\frac{f}{2} & \phi_{2}\phi_{1}\frac{f}{2} & 0 & 0 & 0\\ 0 & 0 & 0 & \phi_{2} & 0 & 0\\ 0 & 0 & 0 & 0 & \phi_{2} & \phi_{2} \end{array}\right]$$ (4)

In our hierarchical population model, three vital rates entered the transition matrix **A**_t_ described above. First, annual fecundity *f* is given by the number of calves produced per female alive just prior to the calving season in spring. Second, juvenile survival (φ_1_) is the first summer survival of calves, with summer here defined as the period between the pre-harvest samples and post-harvest samples taken just after the hunting season in October. Third, annual survival (φ_2_) is estimated for the period between harvest season in year t (represented by *X*_*t*_) until harvest season in year *t+1* (represented by *N*_*t+1*_). For simplicity, we assumed that the latter was the same across sex- and age classes.

### Combining the state- and observation process models

In contrast to most studies where independent estimates of demographic rates are used to parameterize the transition matrix **A**_t_ we were confined to simultaneously estimate latent demographic rates from the data described above. As the different data sets contained information about population structure and/or abundance from different times of the year, the formulations need to be tailored to capture the seasonal changes in structure that might be present as a result of age- and sex differences in survival probabilities.

To model the transition from *X*_*t*_ to *N*_*t+1*_, we specified two equations for yearlings:

$$E(N_{y,t+1}^{i}|X_{c,t}^{i})=X_{c,t}^{i}*\phi_{2}$$ (5)

and for adults:

$$E(N_{ad,t+1}^{i}|X_{y,t}^{i},X_{ad,t}^{i})=[X_{y,t}^{i}+X_{ad,t}^{i}]*\phi_{2}$$ (6)

Superscript *i* = sex (F or M respectively).

Because φ_2_ represents a survival probability that is bound between 0 and 1, we used a binomial distribution to model the survival to ensure that *N*^*i*^_*j*,*t+1*_ (subscript j representing age-class yearling or adult) is an integer number between 0 (if no individual survives) and *X*_*i*_ if all individuals survive:

$$N_{j,t+1}^{i}∼Binom(X_{j,t}^{i},\phi_{2})$$ (7),

where subscript *j* represents age class yearling or adult, respectively.

To model the number of recruits we used the same approach as above, with the probability of success given by the product φ_1_φ_2_*f* = *R*. As reindeer have a maximum littersize of one calf we again used a binomial distribution:

$$N_{c,t+1}^{i}∼Binom(X_{ad,t}^{F},R_{t})$$ (8)

To estimate the parameters in the transition matrix (f, φ_1_ and φ_2_, respectively), we relied on the observation data as described in previous sections. In particular, these data contains information about the sex- and age structure of the populations.

#### PRE-survey

These observations are taken in the spring post-calving, and are related to the *pre-harvest* population vector (*N*_*t*_). Two classes are recognized: calves (of the year) and females together with yearlings of both sexes (hereafter FY). To accommodate the modeling, we made some simplifying but still realistic assumptions. First, we assume that the mortality of FY are minimal through the summer, and that the number of individuals in these age classes in the spring could be approximated by the pre-harvest population vector *N*_*t*_. This assumption is strongly supported by the fact that adult survival in ungulates is generally high and stable in the absence of predation and harvest [34, 38]. Survival probabilities of juveniles might however display considerable temporal and spatial variation [38], and this variation needs to be modeled. We thus allowed for temporal variation in φ_1_ as described above, and modeled the number of calves in the population when the surveys were taken (Calf_t_) by removing φ_1_ from the *R*-function (Eq 8);

$$E({Calf}_{t}|X_{y,t-1}^{F},X_{ad,t-1}^{F})=[X_{y,t-1}^{F}+X_{ad,t}^{F}-1]*[\phi_{2}*f]$$ (9)

We assumed that calves and FY had the same probability of being observed, and specified the observation process through the combination of two binomial probability models;

$$C_{t}^{calf}∼Binom({Calf}_{t},p_{1,t})$$ (10)

$$C_{t}^{FY}∼Binom({FY}_{t},p_{1,t})$$ (11)

Where C_t_^calf^ and C_t_^FY^ is the number of calves and FY observed, respectively, and *Calf*_*t*_ and *FY*_*t*_ is the estimated number in the population at the time of observation, and p_1,t_ is the observation probability assumed to be similar for calves and FY but allowed to vary among years.

#### POST-surveys

After autumn harvest in year *t*, the age- and sex structure of the population is surveyed, and these observations comprise the post-harvest population vector X_*t*_. Lacking independent information about the observation probability (p_2,t_), we made the assumption that p_2_ was similar across age- and sex classes, but we allowed p_2,t_ to vary among years and to be different from p_1,t_. We thus specified the observation process through a combination of three binomial probability models;

$$S_{j,t}^{i}∼Binom(\sum X_{j,t}^{i},p_{2,t})$$ (12)

where *S*_*j*_^*i*^ represents the number of individuals observed in the appropriate sex- and age class. In the post-harvest survey, three classes are identified: calves, females (yearlings and older) and males (yearlings and older). With additional data, deviations in p_1,t_ and p_2,t_between age- and sex classes could have been modeled with the appropriate covariates.

#### TOT-surveys

From aerial line transect surveys we also had access to “minimum count” data, that were sampled in mid-winter in both populations. While the purpose of the sampling is to obtain a minimum count of the reindeer in the area each year, such data are often modelled as if they represent the true state of the population [20]. Count data typically underestimates the true abundance and that variation in detectability will mask true changes in abundance, we included these data as if they represented a sample from the population as use of unadjusted count data is a common practice in state-space models if repeated counts are not available [21]. Acknowledging that the samples are taken post-harvest, the TOT-surveys were included in the model by specifying:

$$Y_{t}∼Pois\left(X_{t}\right)$$ (13),

where *X*_*t*_ is the total abundance and *Y*_*t*_ is the observed number of reindeer in year *t* survey. As noted below we ran models both including and excluding the TOT-data, both on simulated data and on the empirical data from wild reindeer. The model code in JAGS language is available in S1 Code.

### Simulations

To assess the sensitivity of the model performance to violations to model assumptions, we conducted simulation analyses. First, we simulated data according to the above described process, assuming either that all demographic rates (f, φ_1_ and φ_2_, respectively) and the observation probabilities (p_1_ and p_2_) were temporally constant, or that fecundity and juvenile survival as well as observation probabilities p_1_ and p_2_ were varying temporally. We assessed model performance for models including with or without total counts. Then, for the time constant model without total counts, we ran simulations assuming bias (-30% to 30%) in either the post-harvest surveys or in the pre-harvest (spring) surveys. For the latter survey, we assessed two options; male detection probability being biased compared to the other two groups, or female detection probability being biased compared to the other two groups. We simulated time series of 20 years, and for each type of bias considered, we ran 195 simulations (with demographic rates drawn from the distributions in Table 1). Bias was calculated as (biased estimate–true estimate)/true estimate and rescaled to percent bias for presentation.

**Table 1:** Demographic rates used in simulation study.

Vital rate*	Meaning	Range
f	Fecundity	0.60–0.95
φ_1_	Juvenile survival	0.85–0.95
φ_2_	Adult survival	0.88–0.95
*N*_*1*_	Pre-harvest population size in year 1	740–3700

Demographic rates (range) used when simulating data to conduct sensitivity analysis of the hierarchical population model. In all cases, we assumed a uniform distribution bounded by the range.

* N_1_ is not a vital rate.

### Modelling wild reindeer data from Norway

**Fig 1:**
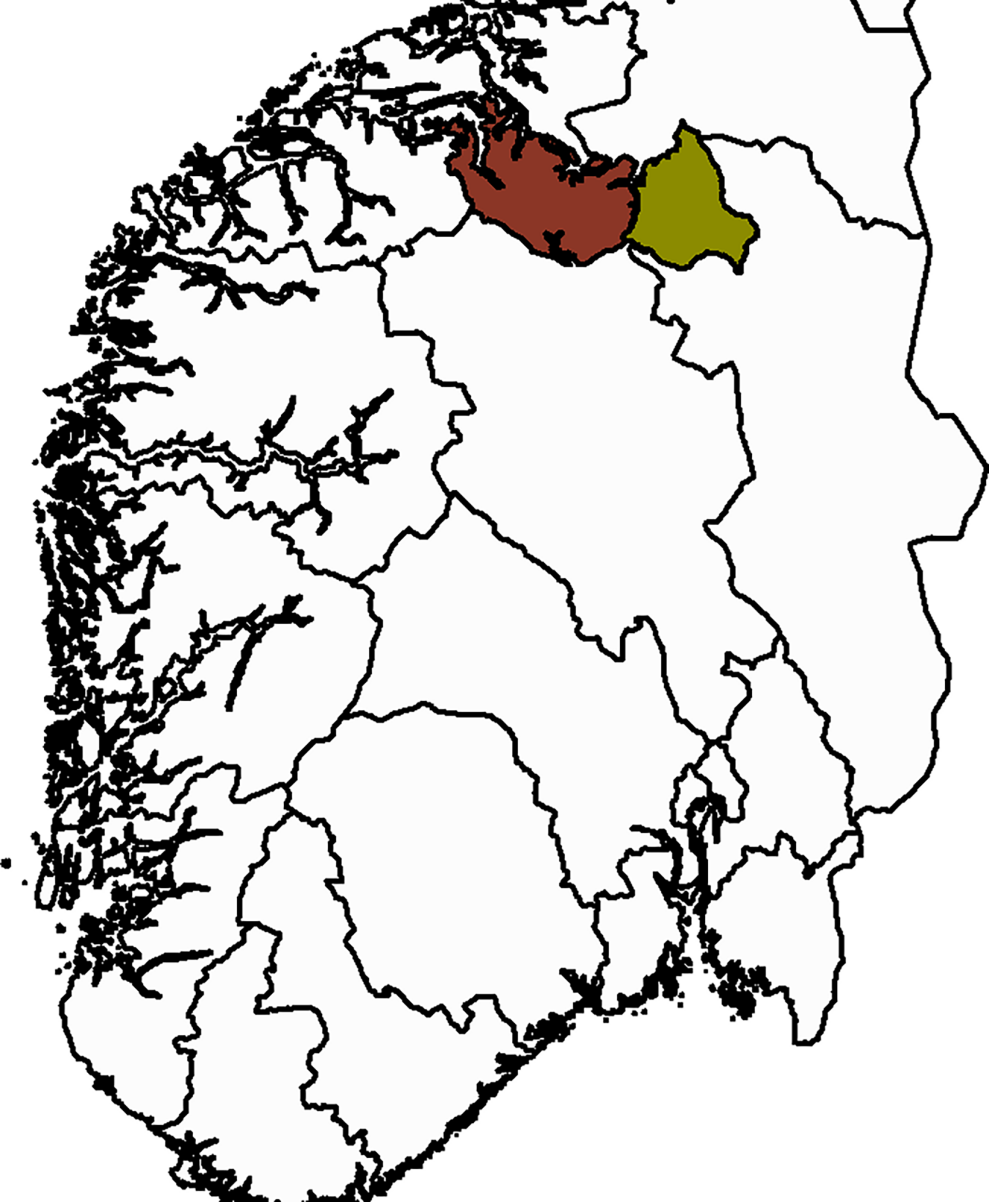
Map showing the location of the study areas. Data for empirical models were collected in the period 1991–2015 as part of the wild reindeer monitoring program in Norway. In the current study, we have used data from Snøhetta (red color on map) and Knutshø (yellow color on map) reindeer areas.

After having assessed the general robustness of our statistical model, we fitted the models to empirical data from two Norwegian wild reindeer populations for a 25-year period from 1991–2015. The two areas, Knutshø and Snøhetta (Fig 1), are both located within the larger Dovre-Rondane wild reindeer region [39]. The areas span a range of geographical gradients, but the main reindeer habitats are occured above the tree line. While the two populations were formerly continuous, major roads and railways have subdivided the area and reduced connectivity between them [18, 39]. Monitoring of wild reindeer in these two populations includes surveys of age- and sex structure in the early summer and in the fall after the hunting season. In addition, detailed harvest statistics (separated in age- and sex groups) are reported by the hunters. In addition, called “minimum counts” [20], are conducted in winter. Data used to fit the empirical models are available in S2 Code.

For each of the two populations, we compared two models: M1 including the TOT-data (i.e. “minimum count” data), and M2 excluding the TOT data. We then compared population trajectories and demographic rates for these two models. In both models, we allowed p_1_ and p_2_ to be time variable, whereas φ_2_ was assumed to be constant. *f* and φ_1_ were modelled as random effects logistic regression models of the form:

$$logit\left(f\left(t\right)\right)=\alpha^{f}+\beta_{t}^{f}$$ (14)

$$\beta_{t}^{f}∼Norm\left(0,\sigma_{f}^{2}\right)$$ (15),

for *f*(*t*) and

$$logit\left(\phi_{1}\left(t\right)\right)=\alpha^{\phi_{1}}+\beta_{t}^{\phi_{1}}$$ (16)

$$\beta_{t}^{\phi_{1}}∼Norm\left(0,\sigma_{\phi_{1}}^{2}\right)$$ (17)

for φ_t_(t). In both cases, α represents the mean rate (on the logit-scale), β is the year-effect, and σ^2^_*f*_ is the variation of the normal distribution from which the year effects are drawn.

### Fitting the hierarchical models

We used Bayesian estimation to calculate demographic parameters and population vectors based on a joint likelihood model (*L*_*joint*_) composed of three independent elements:

$$L_{joint}=L_{TOT}x\;L_{PRE}\;x\;L_{POST}$$ (18)

Where *L*_*TOT*_ is the likelihood for the minimum count data, *L*_*PRE*_ is the likelihood of the recruitment data, and *L*_POST_ is the likelihood of the structural count data. We could also have included a model for the age-at-harvest data, but in our case the harvest bag data are very accurate, and although there is considerable uncertainty regarding the exact age of the harvested animals this should not be of concern when only three age classes are considered.

To estimate the parameters of the hierarchical model, we ran *jags* from R using the add-on library *R2jags* [40]. For the simulated data, we ran 3 chains of 50,000 iterations, with a burn-in of 25,000, whereas we used 150,000 iterations and a burn in of 50,000 to estimate the parameters of the model from the empirical data. The chains were thinned by 3. We specified uninformative priors, using uniform distributions (0–1) for probabilities, and very wide normal distributions (precision-parameter *tau* = 0.0001) for initial population sizes. Convergence was assessed by visual inspection of MCMC-chains and the Gelman-Rubin statistics $\hat{R}$. All data and code developed for this study is available at GitHub (https://github.com/ErlendNilsen/HierarchicalChangeRatio.git).

## Results

### Fitting the models to simulated data

**Fig 2:**
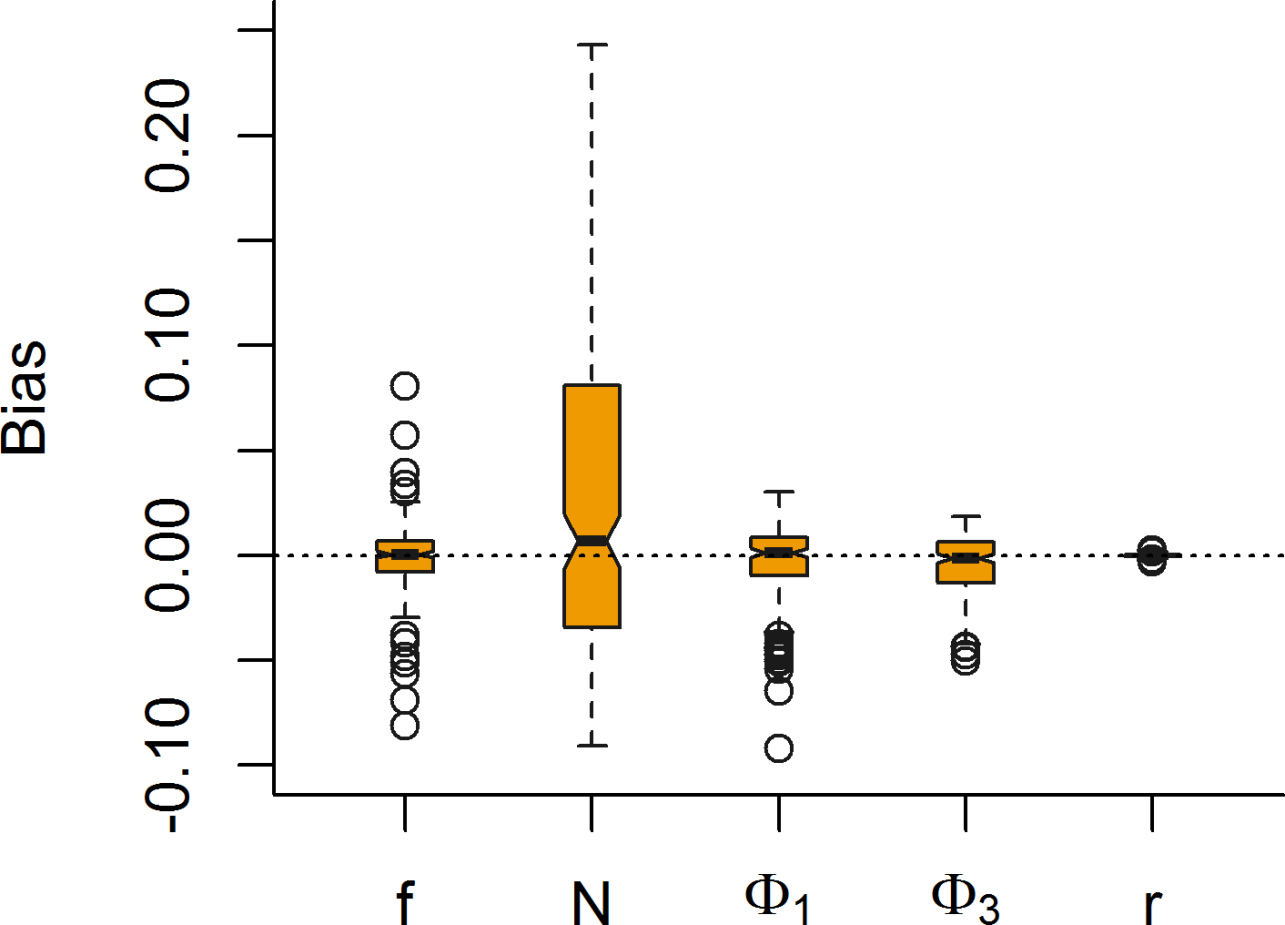
Bias and precision of estimated parameters. Estimated biases in demographic, population size and population growth rate resulting from fitting the hierarchical population model to 200 simulated data sets assuming no bias in the PRE- and POST-harvest surveys of population age- and sex structure. Demographic rates were drawn at random from the distributions in Table 1.

When fitting the population models to simulated data, the population size, population growth rate and demographic rates were effectively estimated regardless if unbiased count data were included or not. This was true when demographic rates were constant through time, and when they were time varying. To assess the bias in the parameter estimates, we simulated 200 data sets of population- and observation data based on the model with time constant detection and demographic rates, with demographic rates drawn from the distributions in Table 1. When fitting the hierarchical model to these simulated data sets, no bias resulting from the hierarchical model was apparent with mean bias <0.4% for both population growth rate and all demographic rates (Fig 2). Concerning estimates of population size, the mean bias was also <2% but the precision for any one simulation was far larger than that of the other parameters of interest (Fig 2). Thus, with access to reliable and unbiased surveys of the population structure, the hierarchical population model developed here provides robust estimates of demographic rates, abundance and population growth rate.

### Sensitivity analysis: Assessing the effects of biased survey data

**Fig 3:**
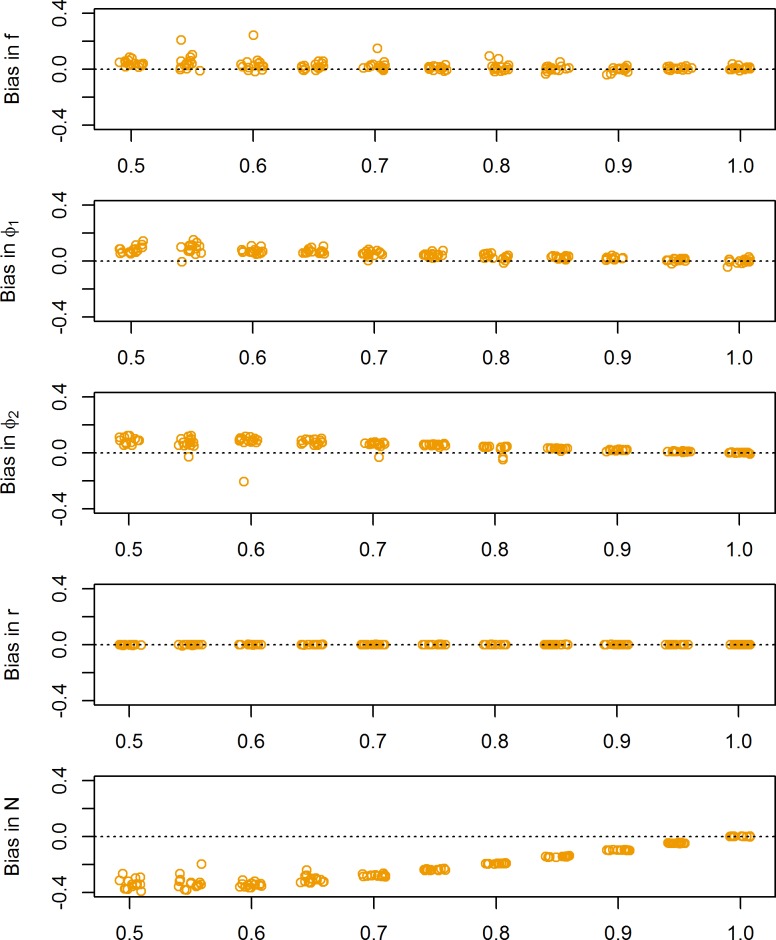
Bias due to biased count data. Effects on demographic rate estimates, population growth rate and population size estimates when fitting the hierarchical including biased TOT-data (i.e. total count data). x-axis indicates the observation probability for the TOT-data. Demographic rates were drawn at random from the distributions in Table 1.

If the estimates of population size from transect counts (TOT-data) underestimated the true abundance, this also resulted in biased estimates of population size (Fig 3). However, such a bias also affected estimates of the demographic rates. In general, this resulted in a slight overestimation of *f*, but the effect was moderate and the bias was <5% as long as the observation probability was >0.55. Similarly, there was a tendency for φ_1_ to be biased high but the effect was relatively small and the bias was <5% as long as the observation probability was > 65–70%. The effect on φ_2_ was comparable, but the magnitude of the bias was slightly larger and the bias was be <5% if the detection probability was >75%.

**Fig 4:**
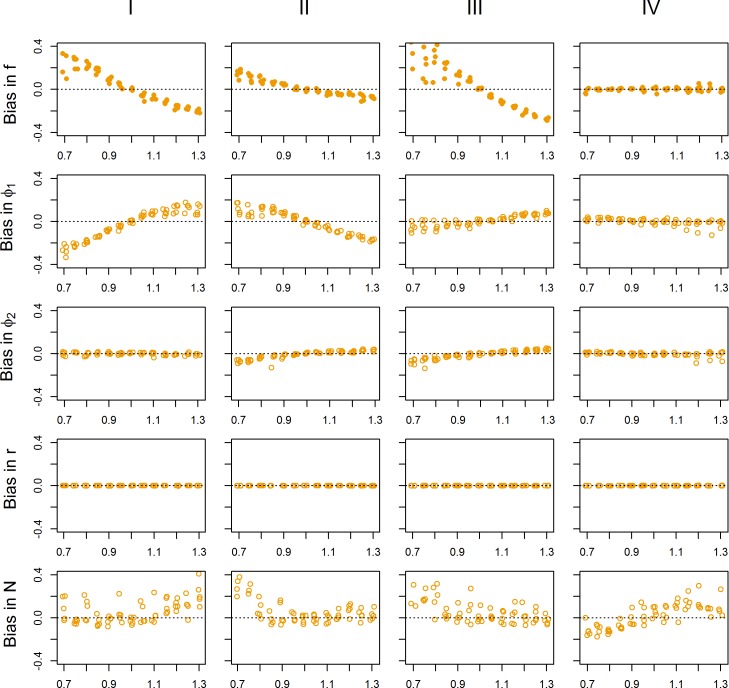
Sensitivity analysis. Sensitivity analysis examining the effects of unequal detection probability among age- and sex classes on estimated model parameters. Roman numbers (I–IV) refers to I) FY detection rate bias relative to calf detection rate in pre-harvest survey, II) female detection rate in relation to calf–and male detection rate in post-harvest surveys, III) FY (pre-harvest) and female (post-harvest) detection rate relative to other classes in the post-harvest samples, and IV) male detection rate relative to calves and females in post-harvest samples. Demographic rates were drawn at random from the distributions in Table 1.

Fitting the models without including TOT-data (i.e. total count data), we assessed the effects of biases in the data from surveys of population age- and sex structure. In general, if males had a different detectability than females and calves in the POST-harvest survey data, it had a negligible effect on the estimated demographic rates (Panel IV in Fig 4). For all demographic rates, the bias in the parameter of interest was <5% for the full range of biases in the survey data considered here (i.e. male detectability being between -70% and 130% that of females and calves). However, if females and yearlings (FY in PRE-surveys) had a higher detectability than calves of the year, *f* was overestimated and φ_1_ was underestimated, with an opposite pattern emerging if FY were more detectable than calves in the spring surveys (Panel I in Fig 4). The same pattern emerged when such a bias is combined with females having different detectability than males and calves in the POST-harvest surveys (Panel III in Fig 4). If the assumption of equal detectability was not met in either PRE- or both PRE and POST-harvest surveys, a bias of <5% in *f* was found when the bias was ≤ 5% in the survey data. If only the PRE-harvest data was biased, the bias in φ_1_ was <5% if the detectability of FY was between 95% and 105% that of calves. However, when both surveys were biased φ_1_ appeared less sensitive, and the bias in φ_1_ was <5% when the bias between -30% and 20%. If only POST-harvest surveys were biased with females having a different detectability than males and calves, the bias in *f* was <5% if the detectability of females is between 85% and 115% that of calves and males (Panel II in Fig 4). Similarly, the bias in φ_1_ was <5% if the bias in the POST-harvest survey data was ≤ ±10%. In contrast to *f* and φ_1_, annual survival φ_2_ was only weekly affected by the types of biases considered above (Fig 4). If there were bias in either the POST-harvest data or both the PRE- and POST-harvest survey data and females (POST-harvest data) or FY (PRE-harvest data) had <80% detection probability compared to the other groups of individuals (i.e. females being 80% that of the other classes) was the bias in φ_2_ >5%. Population growth rate (λ) seemed to be almost unaffected by violations of the equal detectability assumptions, at least within the range of biases considered here. Finally, the bias in the estimated population size (*N*) was >5% when FY had >115% times the detectability of calves in the PRE-harvest survey data, or if females had >85% times the detectability of males and calves in the POST-harvest survey data. If both the PRE- and the POST-harvest survey data were biased, the bias in *N* was >5% when the detection probability of FY and females were <85% that of the other groups. If males had a different detection probability than calves and females in the POST-harvest survey data, the bias in *N* was >5% when males had <80% detection probability compared to the other groups (with *N* biased low) and when males had >110% detection probability compared to the other groups (with *N* biased high).

### Fitting the models to real data from wild reindeer in Norway

Having explored the general properties of our hierarchical population model through simulations, we fitted the model to real data from two populations of wild reindeer in south-eastern Norway (Fig 1). We fitted two versions of the models (M1: including TOT-data from winter, M2: excluding TOT-data). In both models, we assumed that φ_2_ was constant through time, whereas *f* and φ_1_ were modelled using a mixed effects logistic model using year as a random factor to account for year effects.

**Fig 5:**
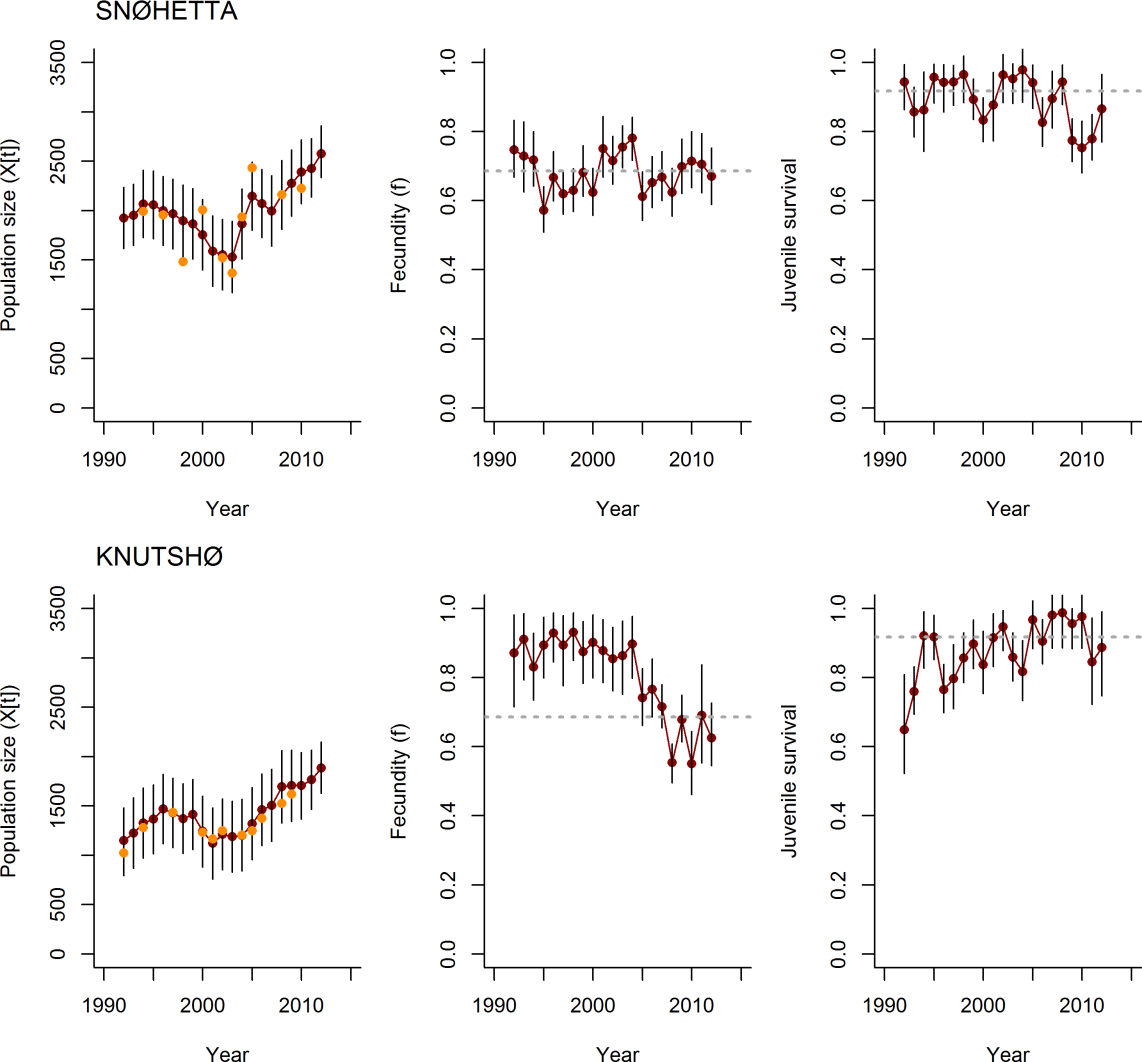
Estimated population trajectories. Estimated time series of abundance (left panels), fecundity (f) (middle panels) and juvenile summer survival probability (φ_1_) (right panels) for Snøhetta (upper panels) and Knutshø (bottom panels) respectively. Model output is based on M1 (see Methods section).

**Fig 6:**
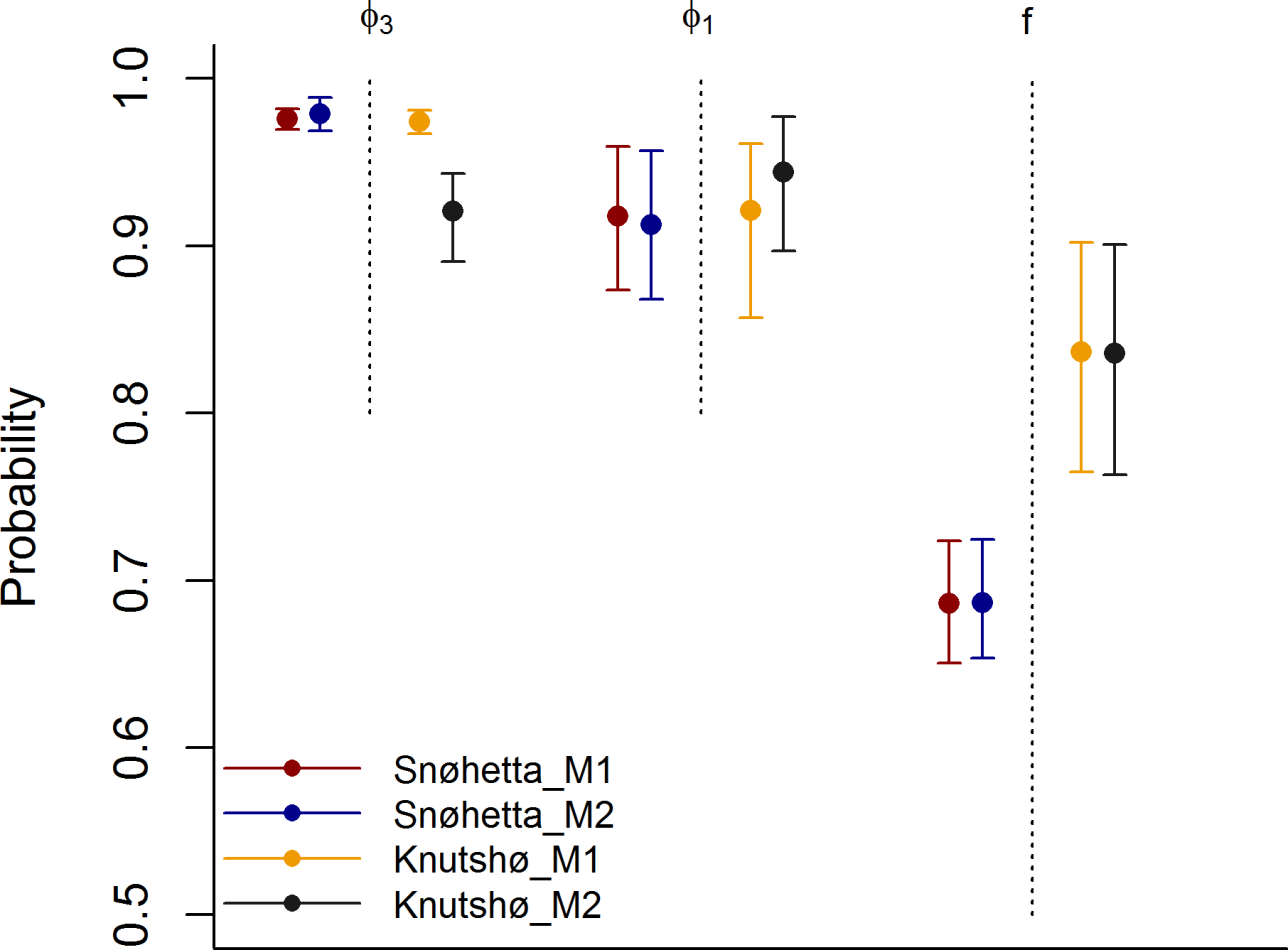
Comparison of M1 and M2. Comparison of estimated demographic rates based on M1 and M2 for Snøhetta and Knutshø, respectively. Note that f and φ_1_ are back-transformed from logit-scale and thus represents true probabilities. The two latter were estimated using mixed effects logistic regression models outlined in Eqs 11–14.

In Snøhetta, M1 (assuming TOT-data sampled from a Poisson distribution) and M2 (excluding TOT-data) produced similar estimates of mean fecundity *f* (M1: 0.64 [0.58–0.70, 95% Bayesian c.i.], M2: 0.64 [0.60–0.70, 95% Bayesian c.i.]) and juvenile survival φ_1_ (M1: 0.94 [0.92–0.97, 95% c.i.], M2: 0.95 [0.91–0.98, 95% c.i.]) (Figs 5 and 6). The estimated annual survival of adults φ_2_ was greater in model M1 (0.97 [0.96–0.98]) compared to M2 (0.94 [0.93–0.96]). This might reflect the fact that the minimum counts underestimated true abundance, and a similar pattern was found when we analyzed simulated data with minimum counts biased low. The population growth rate λ (through the mean estimated population sizes) was estimated at 1.00 (95% C.I: 0.99–1.01) for M1 and 1.00 (95% C.I: 0.99–1.00) for M2.

In Knutshø the most marked difference in estimated demographic parameters between the two models was for φ_2_ (M1: 0.97 [0.96–0.98]; M2: 0.93 [0.90–0.95]). Similar to Snøhetta, the mean fecundity *f* (M1: 0.87 [0.81–0.93], M2: 0.87 [0.81–0.93]) and mean juvenile survival φ_1_ (M1: 0.91 [0.82–0.96], M2: 0.93 [0.87–0.97]) were similar for the two models (Figs 5 and 6) in Knutshø. The population growth rate λ (through the mean estimated population sizes) was estimated at 1.01 (95% C.I: 1.00–1.03) for M1 and 1.02 (95% C.I: 1.01–1.03) for M2.

## Discussion

We first formulated a hierarchical population model based on total counts and data on population age- and sex structure, and change-in-ratio after harvest. Then we conducted extensive sensitivity analyses based on simulated data, and fitted the model to empirical data from a wild reindeer monitoring program in Norway. Based on our sensitivity analyses we conclude that the approach developed here is able to obtain precise estimates of demographic rates whenever unbiased data of population structure is available. Our simulations revealed that this was true also when population abundance is not available or not included in the modelling framework. Nevertheless, we show that when the modelling is based on biased population structure data due to different observability of different age- and sex categories it will affect estimates of all demographic rates. In particular, estimated population size sensitive to such biases.

When modelling real data from reindeer in Norway, we compared model output from two versions of the model (i.e. with or without TOT-data). We found that the estimated fecundity (f), juvenile summer survival (φ_1_) and annual survival (φ_2_) were virtually unaffected by the choice of model. This result is reassuring in cases when estimating such rates are the main purpose of the study- or monitoring program. The challenge, of course, is that while we are able to directly assess bias and precision for the simulated data, this is not possible for the real data as we do not have access to independent data sources. However, comparing estimated demographic rates to published values for long-lived medium-sized ungulates [41–43] suggest that the rates reported here are not severely biased. Also, based on the simulation study (Fig 4), annual survival (φ_2_) seems to be relatively unaffected by biases in detection probabilities among age- and sex classes as long as these are consistent across years. A similar conclusion was drawn by O`Brien et al. [44] when assessing the consequences of violating assumptions in mark-recapture models. Further, if the TOT-data is biased low (which might be the case if not all animals are detected), φ_2_ will be overestimated. However, as long as detection probability is >75% the bias will be less than 5%. Comparing M1 and M2 for the Snøhetta and Knutshø populations respectively, the relative difference between estimated φ_2_ from the two models were <1% (Snøhetta) and 5% (Knutshø) respectively. For the other rates, the difference between M1 and M2 was negligible.

In our example of wild reindeer in Norway, we used count data for total annual numbers of reindeer of different age- and sex categories as a basis for our models. We suggest that having access to raw observation-data will increase flexibility, and can potentially allow for more accurate modelling of the observation process. Explicit modelling detection as function of covariates could help in correcting for biases due to uneven detection probabilities among sex- and age classes. We thus recommend that the monitoring program adjust their data handling protocol to make such data readily available. The sensitivity analysis indicated the importance of modelling *p* as a function of age and sex in reducing the potential biases in these estimates. Here, the potential sources for biased or uneven detectability between age- and sex groups must be identified. In our case uneven detectability might arise if there is spatial segregation between age- and sex classes (see e.g. [45] for red deer *Cervus elaphus*) that are not accounted for in the study design, if e.g. calves are less observable because of their smaller size, or if observers are more likely to assign given age- or sex to animals where certain assignment is difficult based on morphological characters (e.g. between adult females and yearlings of both sexes in spring).

Monitoring programs for ungulates and large carnivores usually span large spatial scales due to high mobility of animals. Thus, identifying methods than be applied across large spatial and temporal scales is key to future sustainable management. Often monitoring programs in these cases are based on indices or “minimum counts” with no possibility to assess accuracy and potential biases inherent in the programs [46]. For instance, in the study by Popescu et al. [46], growth rates based on replicated population counts were compared with biological plausible growth rates based on reported demographic rates for brown bear, wolf (*Canis lupus*) and Eurasian lynx (*Lynx lynx*). While such comparison of monitoring data from data-poor systems with more robust demographic data to assess the biological plausibility of the observed data provides information about potential pitfalls related to relying solely on such data, the approach taken in our study suggest that in many cases combining the data sets afford greater opportunity to maximize inference from a variety of sources.

We are not the first to advocate hierarchical models combining information from different data sets to gain increased insight into demographic processes and to obtain better estimates of abundance [47]. In fact, the motivation for developing integrated population models, as discussed in e.g. [47–49], is to maximize inference from available data. Our work thus clearly benefits from the many previous studies within this field, and the novelty in our work is not so much that we combine data from multiple sources but rather the particular setting and type of data that we use as a basis for our modelling. In general, we have here shown how change-in-ratio models [26] can be extended using time series data allowing the full temporal dynamics to be modelled. We believe this extension should be useful in many natural resource issues.

In summary, we suggest that the approach developed here can provide managers with unbiased estimates of demographic rates (i.e. survival and reproduction) and population abundance in cases where time series data on population structure data are available. Sampling auxiliary data and making sure the study design allows for estimation of different detectability between age- and sex groups should be prioritized. Our sensitivity analyses suggest that monitoring changes in demographic rates can be a more sensible approach when direct surveys of population size cannot be conducted but managers do have access to data describing changes in age- and sex structure of a wildlife population through time. The hierarchical models we present here are based on time series data and in principle allow a full temporal decomposition of the population dynamics, which should greatly extend the usefulness of such data.

## Supporting information

S1 Code JAGS code detailing the code describing the integrated hierarchical change in ratio models (R). (R)

S2 Code R code used to simulate data, used in sensitivity analyses and to generate initial values for model runs. (R)

S3 Code R code used to execute and run the JAGS code in appendix 1, based on the empirical data provided in S1 Spreadsheet. (R)

S1 Spreadsheet Data from the reindeer monitoring used to fit the change-in-ratio models. (CSV)

S1 Text Text file detailing the data content in S1 Spreadsheet. (TXT)
